# Cardiac Arrest Recovery Enablement and Supported Self-management (CARESS): a study protocol for the feasibility testing of an online psychosocial and exercise rehabilitation programme for cardiac arrest survivors and co-survivors

**DOI:** 10.1016/j.resplu.2026.101270

**Published:** 2026-02-12

**Authors:** N.A. Pearson, G. McGregor, H. Sandhu, K. Couper, J. Bruce, P. Swindell, S. Menzies, D.R. Ellard, R. Kandiyali, S. Ennis, S. Patel, A. Hossain, K.L. Haywood, CARESS Team, Paul Swindell, Stuart Menzies, Jeanne Reilly, Anne Brooks, Dave Cleland, Bernie Cleland, Asad Kayani, Rakhsana Yousaf, John Long, David Ellard

**Affiliations:** a University Hospitals Coventry and Warwickshire NHS Trust, Coventry CV2 2DX; b Warwick Medical School, University of Warwick, Coventry CV4 7AL; c Critical Care Unit, University Hospitals Birmingham NHS Foundation Trust, Birmingham, UK; d Patient and Public Involvement and Engagement (PPIE) partner

**Keywords:** Cardiac arrest, Survivorship, Recovery, Survivors, Co-survivors, Self-management, Behavioural interventions

## Abstract

**Aim:**

Little is known about how best to deliver support for the long-term recovery of cardiac arrest survivors, their family and close friends (co-survivors), or whether providing structured support is feasible. Working collaboratively, we have co-produced two online psychosocial and exercise rehabilitation care pathways for adult survivors and co-survivors – the Cardiac Arrest Recovery Enablement and Supported Self-management (CARESS) intervention. This study assesses the feasibility and acceptability of patient identification, recruitment, intervention delivery and outcomes assessment.

**Method:**

A single arm, multi-centre feasibility study with an embedded process evaluation. We will test the acceptability and delivery of a new rehabilitation intervention in the NHS setting. We will recruit 30 cardiac arrest survivors discharged to home, and 30 co-survivors.

The CARESS rehabilitation intervention consists of three components delivered in real-time by video-call:

1. One-to-one consultation with a CARESS facilitator (week 1): a one-hour conversation to discuss motivation and set goals.
2. Facilitator-led group support sessions (weeks 2–8; 1-h): provided weekly, separately for survivors *or* co-survivors to develop their knowledge, skills, understanding and establish connections with peers.
3. Supervised group exercise rehabilitation sessions (weeks 3–8; up to 45 min): provided weekly, for survivors to build confidence and fitness.

Recruitment, retention, and completion of outcome measures will be evaluated. Exploratory outcomes will include health-related quality of life (PROPr 29+2), emotional wellbeing (HADS, WEMWBS), and fatigue (FACIT-f), measured at baseline (week 0) and post-intervention (week 11) to assess suitability for a randomised controlled trial. Interviews to explore participant and facilitator perspectives will inform the process evaluation.

**Discussion:**

This study assesses the feasibility of online delivery and findings will inform a future multi-centre randomised trial.

**Trial registration:** ISRCTN16382742, prospectively registered 28/01/2025.

## Introduction

Advances in resuscitation science across the ‘chain of survival’ have resulted in a growing number of cardiac arrest survivors,^1^, ^2^, ^3^ with average survival rates at hospital discharge in Europe now approximating 8% (range 0–18%).^4^ However, mounting evidence details the substantial and enduring health challenges and associated needs experienced by survivors and their families on their recovery journey.^1^, ^2^, ^5^ Both the cardiac event and subsequent treatments can contribute to predominantly negative long-term psychological, cognitive, physical, and social consequences.^1^, ^2^, ^3^, ^6^, ^7^, ^8^ There is often a marked emotional impact on immediate family members and close friends (herein, referred to as co-survivors).^1^, ^6^, ^9^, ^10^, ^11^

Enhancing recovery and survivorship following cardiac arrest are key challenges for the international resuscitation community,^1^ with recovery increasingly recognised as an essential link in the survival chain.^12^ Recent guidelines^3^, ^12^ and quality standards,^13^, ^14^ promote more formalised, tailored support for survivors and co-survivors. Early intervention, commencing within the first three-months post-arrest, and preferably starting from hospital discharge, is recommended. However, for many, support after hospital discharge remains inadequate.^1^, ^15^, ^7^, ^8^, ^9^, ^10^, ^11^ There is uncertainty around care optimisation and evidence informing rehabilitation pathways for this vulnerable group is scarce.^1^, ^2^, ^3^, ^6^, ^9^, ^12^, ^16^, ^17^, ^18^ High quality trials comparing rehabilitation to standard care alone in cardiac arrest survivors are urgently required.^18^ The paucity of co-survivor focused interventions underpins the need to prioritise co-survivor interventions to improve health and wellbeing.^19^

However, structured rehabilitation interventions designed to address the physical and emotional health needs of people living with other chronic conditions, such as long-COVID^20^ and pulmonary hypertension,^21^ are helpful. Designed to improve psychosocial and physical health, these complex interventions address symptoms that overlap with those experienced by cardiac arrest survivors, including anxiety, fear avoidance of activity, cognitive impairment, fatigue, muscle weakness, and reduced mobility. Taking advantage of recent shifts in the acceptability of virtual healthcare, successful online delivery of these supervised, home-based group interventions has also been described.^20^, ^21^, ^22^ Given the relatively small number of cardiac arrest survivors and co-survivors in any one geographical region, centralised online delivery has the potential to increase accessibility to group-based interventions for people with similar ‘lived experiences’, a consideration that is highly valued by cardiac arrest survivors and their co-survivors.^5^, ^19^

Building on existing evidence, we developed a new rehabilitation programme, co-developed with public partners. The Cardiac Arrest Recovery Enablement and Self-management Support (CARESS) intervention is a structured programme suitable for online, facilitator-led and group-based rehabilitation designed to address the psychosocial and physical needs of cardiac arrest survivors and co-survivors.

### Aim

To test the feasibility of delivering CARESS to adult survivors and co-survivors in the NHS setting. This will be achieved through the following objectives:

1. Assess the feasibility of recruiting survivors and co-survivors from hospital settings or other self-referral methods within 12-months of their cardiac arrest by evaluating the:
  • ability to identify and recruit eligible people (proportion of eligible survivors/co-survivors invited and/or recruited).
  • process of gaining digital consent.
2. Assess the acceptability and feasibility of the CARESS intervention.
  • Iteratively refine the intervention and facilitator training manuals.
  • Assess the quality of intervention delivery procedures and if delivery was as intended. This was informed by existing delivery methods for comparable complex interventions.^20^, ^21^, ^23^
3. Assess acceptability and feasibility of data collection procedures by evaluating the:
  • acceptability, completeness and respondent burden of digital data collection.
  • distribution properties, mean and standard deviation of the PROMIS 29+2 score (proposed future primary outcome) to inform sample size requirements for a future randomised controlled trial. Analysis will also include evaluating missing data, completion rates, floor and ceiling effects and assessing standard deviations per domain.

## Method/design

A single-arm feasibility study with an embedded process evaluation (Table 1). Informed by similar feasibility studies^24^, ^25^ and current practice,^26^ we will recruit 30 survivors and 30 co-survivors. Participants will be recruited simultaneously from up to three NHS Trusts serving diverse populations across the West Midlands and South-West of England. Self-referral will be facilitated through national charities including Sudden Cardiac Arrest UK (https://suddencardiacarrestuk.org) and Air Ambulance services. The protocol is structured and reported using the Standard Protocol Items: Recommendations for Interventional Trials (SPIRIT) checklist.^27^

**Table 1:** Summary of the CARESS feasibility study.

Primary registry and trial identifying number	ISRCTN16382742
Date of registration in primary registry	January 28, 2025
Secondary identifying numbers	REC 24/NE/0135 NIHR204049
Funder	National Institute for Health and social care Research (NIHR) – Research for Patient Benefit (tier two) The funder has no role in study design, data collection, analysis, publications or dissemination.
Sponsor	University of Warwick University House Annex Coventry, CV4 7AL sponsorship@warwick.ac.uk The sponsor has no role in study design, data collection, analysis, publications or dissemination.
Contact for public queries	UHCW NHS Trust University Hospital Clifford Bridge Road Coventry, CV2 2DX nathan.pearson@uhcw.nhs.uk
Contact for scientific queries	University of Warwick Warwick Medical School Gibbet Hill Road Coventry, CV4 7AL k.L.haywood@warwick.ac.uk
Public Title	Cardiac Arrest Recovery Enablement and Supported Self-management (CARESS) feasibility study
Scientific title	Cardiac Arrest Recovery Enablement and Supported Self-management (CARESS) feasibility study
Countries of recruitment	England
Clinical Phase	Feasibility study
Health condition(s) or problem(s) studied	Cardiac arrest
Intervention(s)	1. Individual assessment (week 1) 2. Online group support sessions (weeks 2–8) 3. Exercise rehabilitation (survivors only, weeks 3–8)
Key eligibility criteria	**Survivors** Inclusion: adult (aged 18+ years) survivors of in-hospital or out-of-hospital cardiac arrests, discharged from hospital within 30 days of the arrest. Recruited within 12-months of their cardiac arrest. Proficient in English and able to complete online follow-up and sessions. Exclusion: Unable to give informed consent or have severe mental health or cognitive impairments preventing engagement. Participating in other support programmes (usual care is permitted). **Co-survivors** Inclusion: adult (aged 18+ years) family member, friend or significant other (‘co-survivor’) of a cardiac arrest survivor. Recruited within 12-months of the survivor’s cardiac arrest. Proficient in English and able to complete online follow-up sessions. Exclusion: Unable to give informed consent or have severe mental health or cognitive impairments preventing engagement. Participating in other support programmes or a family member or friend of someone who did not survive.
Study type	Multi-centre single-arm feasibility study with an embedded process evaluation
Date of first enrolment	July 10, 2025
Target sample size	60 (30 survivors, 30 co-survivors)
Recruitment status	Recruitment launched June 20, 2025
Primary outcome(s)	Feasibility and process-related measures: rates or recruitment and retention (numbers screened, eligible, recruited); outcome measurement (acceptability, respondent burden, completion rates, missing data); intervention quality; acceptability of intervention and study processes (process evaluation interviews).
Key secondary outcomes	At baseline (week 0) and post-intervention (week 11): Health-related quality of life (PROPr v2.1); Mental wellbeing (WEMWBS); Fatigue (FACIT-f, PROPr fatigue subscale); Depression and anxiety (HADS, PROPr depression and anxiety subscales); post-traumatic stress disorder symptoms (IES-R); Health utility (EQ-5D-5L). Post-intervention (week 11) only: Health and social care resource use (ModRUM); personal resource use (internally generated questionnaire).

### Participants

30 adult survivors of in-hospital or out-of-hospital cardiac arrests (of ischaemic or idiopathic origin), discharged within 30 days of admission, and recruited within 12-months of the cardiac arrest. 30 adult co-survivors (family member, spouse, close friend or significant other impacted by the arrest) of such survivors recruited within the same timeframe through a survivor nomination, or self-referral. Eligibility criteria are presented in Table 2. Participants will not be prohibited from accessing concomitant care.

**Table 2:** Participant eligibility criteria.

	**Inclusion**	**Exclusion**
All participants	Adult aged 18+ years	Unable to give informed consent
	Able to complete online follow-up and attend online sessions	Have severe mental health difficulties or cognitive impairment that prevents engagement
	Proficient in the English Language	Are participating in another exercise, psychological support, or structured rehabilitation study

Survivors	A survivor of an in-hospital or out-of-hospital cardiac arrest	
	Discharged from hospital to their usual place of residence within 30 days of their cardiac arrest	
	Recruited before discharge, or within 12-months of their cardiac arrest	

Co-survivors	Self-refer into the study or are referred as a nominated adult family member, spouse, close friend or significant other of a cardiac arrest survivor	Family members or close friends of cardiac arrest victims who do not survive to hospital discharge/remain hospitalised beyond 30-days of the arrest
	Recruited within 12-months of the survivor’s cardiac arrest	

### Screening, recruitment, and consent procedures

**Fig. 1:**
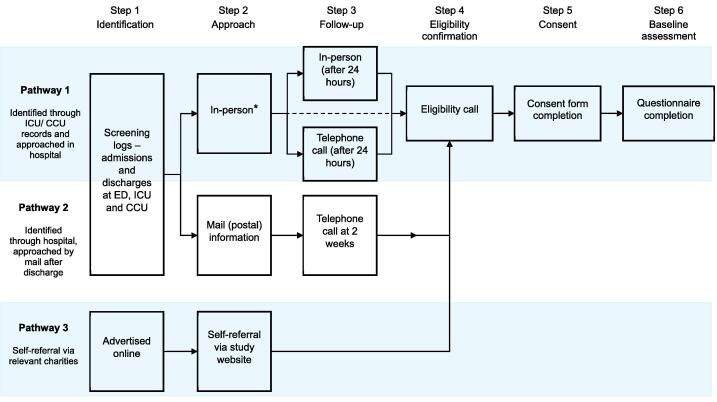
**Recruitment pathways into the CARESS feasibility study**. ICU = Intensive Care Unit; CCU = Coronary Care Unit; ED = Emergency Department; * = the dashed line in pathway 1, steps 2–4 represents people who decide to participate upon approach. They can register interest at that time and thus do not require follow-up.

Three recruitment pathways for survivors and co-survivors (Fig. 1):

1. Identified and approached in-hospital.
2. Identified in hospital and approached post-discharge.
3. Self-referral via social media and/or national charities

#### Pathways 1 & 2 – Hospital-based recruitment

Clinical teams identify eligible participants via intensive care units, coronary care units or emergency departments, cardiac rehabilitation registers, and resuscitation logs. Study materials (flyer, invitation letter, participant information sheet) are given to potential participants) or sent by post and include a link/QR code to the online expression of interest (EOI) form. People who were approached are followed up by the clinical team at least 24-h after initial contact to discuss the study and answer any questions. Follow-up will then be made in person or by phone depending upon pathway and patient preference. Interested individuals will be contacted for a baseline call to confirm eligibility and obtain informed consent. Survivors will complete two cognitive assessments by telephone: the Modified Rankin Scale (mRS)^28^ and Montreal Cognitive Assessment (MoCA).^29^, ^30^ Baseline data will include demographic information and exploratory outcome data collected online via questionnaires.

Co-survivors are identified through survivor nomination or during hospital visits. They receive the same information, follow the same consent process and complete baseline assessments but will not complete cognitive assessments. Reminder follow-up contacts will be made at two and four weeks (telephone call, email, or letter) if no EOI has been returned.

#### Pathway 3 – Self-referral via charities or social media

Charities supportive of the study such as SCAUK and responder services (Air Ambulance) will advertise the study using materials with links/QR codes to the study website and EOI form. Interested individuals who contact the study team will be contacted, screened, consented, and will then follow the same pathway as described above.

We will actively collaborate with our PPIE partners and key stakeholders throughout intervention development and testing, facilitating a patient-centred approach to all activities and interactions. Practical approaches to promote participant retention will be shared across the study team and facilitators.

### The CARESS intervention

CARESS was co-produced, revised and refined based on reviews of published evidence and participant input through in-person workshops, and focus groups. Whilst a separate intervention development paper will follow, we summarise CARESS as a complex intervention^31^ designed to support survivors and their co-survivors over eight weeks, through two distinct pathways tailored to their unique needs.

• The **survivor pathway**: addresses the physical and mental/emotional health needs as people recover from cardiac arrest. The programme centres on building confidence to return to physical activity.
• The **co-survivor pathway:** recognises the emotional toll of the cardiac arrest, particularly if a co-survivor has witnessed the cardiac arrest or provided CPR. The programme centres on addressing co-survivor mental/emotional wellbeing needs.

**Fig. 2:**
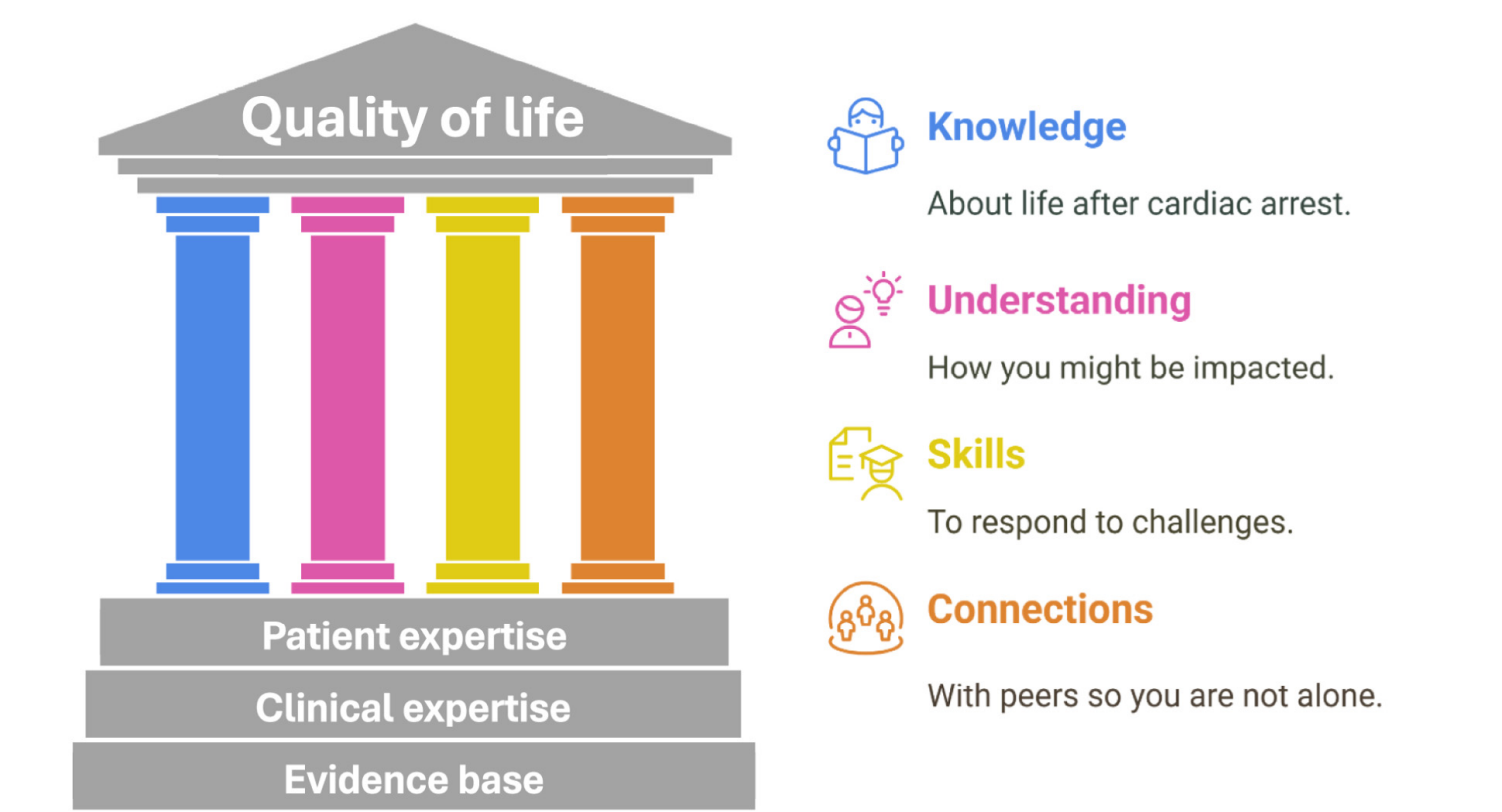
**The four pillars of the CARESS intervention**.

Four competences underpin the CARESS intervention (Fig. 2):

(i) building **knowledge** about cardiac arrest and survivorship;
(ii) developing **understanding** about implications for health and wellbeing;
(iii) providing **skills** to adapt, respond and cope with associated challenges; and
(iv) developing **connections** with others to become part of a peer support network.

CARESS consists of three components: a one-to-one individual assessment (week 1), followed by weekly online group support sessions (weeks 2–8). Survivors also participate in weekly online exercise (weeks 3–8).

#### Component 1: one-to-one individual assessment (week 1)

All survivors and co-survivors complete an individual 1-h appointment with a CARESS facilitator (clinical exercise physiologist). Delivering a holistic assessment of participant needs, this includes: an introduction to the programme; individualised advice; and an opportunity for participants to set goals and discuss any concerns. For survivors, relevant medical information will be gathered to ensure participant safety, particularly regarding exercise. All participants will be directed to freely available external resources, such as those provided by Sudden Cardiac Arrest UK (https://www.SCAUK.org).

#### Component 2: weekly group online support sessions (weeks 2–8)

Core theoretical principles have been drawn on to inform the psychosocial content, structure, and delivery. These include the biopsychosocial model of behaviour change,^32^, ^33^ Michie’s Behaviour Change Wheel and taxonomy,^34^ Michie’s COM-B model (Capability, Opportunity and Motivation), and psychological theories of self-efficacy,^35^ cognitive behaviour-change, and motivational interviewing.^36^ These frameworks supported identification of capability, opportunity, and motivation barriers and enablers to recovery. This informed the intervention components and behaviour change techniques. Capability was targeted through structured self-management skills addressing behavioural, psychological/cognitive and emotional coping (e.g., fatigue management through established pacing techniques). Opportunity was enhanced through facilitated online group delivery and engagement with peer groups. Motivation was supported through goal-focused one-to-one and group discussions. Together, the frameworks guided intervention development, content, structure and delivery.

Survivors and co-survivors will participate in separate weekly online sessions delivered in real-time by video call for seven weeks, with each session lasting up to 1-h. Sessions will be led by a CARESS facilitator, an experienced clinical exercise physiologist with experience in delivering similar psychosocial interventions. Facilitators are trained and supported by health psychologists and intervention experts. The role of facilitators in this session is to share educational information, but primarily to facilitate and stimulate group discussion through open questions.

Participants will have access to the material covered in the group support sessions through a written, printed workbook which is mailed to them. This will allow participants to prepare for or consolidate learning after sessions. Early sessions will focus on motivation and goal setting to support sustained behaviour change, and supplemented by group discussion about opportunities, setbacks and self-reflections.^34^

Each week, a different topic will be discussed with the group. Facilitators will guide participants through the topic with a supporting slide deck, sharing information and skills or techniques to help self-manage potential challenges. Survivor sessions also include short videos of survivors describing how they are impacted by the topic of the week. Throughout the session, participants will be invited to share their experiences and questions will be posed to the group to stimulate group discussion. Participants will be advised to further develop their understanding through engagement with the study workbook.

#### Component 3: weekly online group exercise rehabilitation sessions (weeks 3–8)

This component is only available to cardiac arrest survivors and is delivered in real-time by video call. After the one-to-one and two weeks of attending group online support sessions, individualised exercise rehabilitation will be initiated. Weekly, supervised sessions lasting for up to 45 min will be completed (including warm-up and cool down). Survivors will be advised to supplement this with up to two self-directed exercise sessions per week. We will recommend British Heart Foundation resources for self-directed exercise (https://www.youtube.com/@britishheartfoundation/playlists). This rehabilitation is intended to be light-moderate exercise to build confidence. Exertion during the exercise sessions will be monitored using the talk test.^37^ Warm-up and cool down will be guided by individual needs.

### Feasibility study outcomes

Overall study feasibility and process-related measures:

• *Rates of recruitment and retention across the recruitment window:* captured via screening logs recorded by participating NHS sites at baseline (week 0), 4-months (week 16) and 8-months (week 32). Data will include when someone was identified, approached and can be cross-referenced with who participated. For self-referral, we will gather and assess data on the numbers who express interest, are eligible, consent, and participate.
• *Outcomes measurement:* acceptability, respondent burden (e.g., time to complete, completion rates), and completeness (missing data) assessed using descriptive statistics at baseline and follow-up (week 11) and participant responses during process evaluation interviews.
• *Intervention quality:* assessment of delivery procedures for fidelity with study participants and facilitators measured through structured reviews of recordings from a subset of sessions, guided by current guidance^38^ assessed post-intervention (weeks 9–13)
• *Process evaluation:* assessment of recruiter, participant and facilitator perspectives and experiences of delivery or participation in the study, explored through post-intervention interviews (weeks 9–13)

Exploratory outcomes are described in Table 3. Measurement selection was informed by evidence of acceptable psychometric properties in relevant populations.^39^, ^40^, ^41^, ^42^, ^43^, ^44^, ^45^, ^46^, ^47^

**Table 3:** Exploratory outcomes measured at baseline and post-intervention.

**Patient-reported outcome measure**	**Purpose**	**Assessment time**	
		**Baseline** **(week 0)**	**Post-intervention** **(week 11)**
Patient-Reported Outcomes Measurement Information System (PROMIS®) Preference Scoring (PROPr) 29+2 Profile v2.1 (1)	Summary scores of physical and mental health	✓	✓
Warwick-Edinburgh Mental Wellbeing (WEMWBS) (2)	Mental wellbeing	✓	✓
Functional Assessment of Chronic Illness Therapy – fatigue (FACIT-f) (3)	Fatigue	✓	✓
Hospital Anxiety and Depression Scale (HADS) (4)	Anxiety and depression	✓	✓
Impact of Events Scale-Revised (IES-R) (5)	Post-traumatic stress disorder symptom severity	✓	✓
EuroQoL EQ-5D-5L (6)	Health utility	✓	✓
Modular Resource-Use Measure (ModRUM) core (7)	Health and social care resource use	✗	✓
Internally generated personal resource use questionnaire	Personal resource use	✗	✓

### Data collection and management

Digital case report forms and participant questionnaires will be used to collect all study data. Access to digital identifiable information and study documents will be restricted and held on a secure, password-protected system. Names or addresses of participants will not be disclosed to anyone other than personnel directly involved with study delivery. A unique identifier will be used on documents other than the consent form and postal documents. An approved, GDPR compliant online data capture software (Qualtrics) will be used to gather data from participants. The study will be conducted in accordance with the current approved protocol, Good Clinical Practice, relevant data protection regulations and Sponsor standard operating procedures. Data already collected will be retained for participants who discontinue from the study, unless they withdraw their consent.

Confidentiality will be strictly maintained and names, addresses or personal identifiable information will not be disclosed to anyone other than the staff involved in running the study. All electronic participant-identifiable information will be held on a secure, password-protected excel file accessible only to essential personnel.

### Data analysis

Analyses will be descriptive, summarising feasibility outcomes and assessing the acceptability of CARESS. Inclusivity (age, sex, ethnicity, socioeconomic status) will be assessed for each of the three recruitment pathways. Participant demographics will be summarised using descriptive statistics at baseline and post-intervention (week 11). Findings from mRS and MoCA will be summarised for survivors.

Recruitment and retention rates will be reported at baseline and post-intervention by recruitment pathway. Missing data will be examined at baseline and post-intervention, and characteristics of those who did and did not complete outcome measures will be compared descriptively. The acceptability of recruitment methods and processes will be assessed using descriptive statistics, including recruitment, eligibility and consent rates, retention, intervention attendance and outcome measure completion. Inclusivity of recruitment will be examined descriptively, by pathway comparing characteristics including age, sex, ethnicity and socio-economic status. Qualitative insights about acceptability will be explored through process evaluation interviews.

Exploratory outcomes will be assessed to determine participant burden through completion times, and feedback. Process evaluation interviews will include direct discussion about outcome measurement, preferences, and why. Included in our exploratory outcome measures, we are piloting the acceptability of health economic data collection. We will examine the completion rates, and floor and ceiling effects for the EQ-5D-5L.^48^ We will similarly examine completion rates of healthcare resource use data collected using the ModRUM core module.^49^ Completion rates of a social care and personal resource use – captured using a survivor and co-survivor co-produced questionnaire – will also be examined.

### Process evaluation (embedded interview study)

This phase of work draws on the MRC guidance for process evaluations of complex interventions to explore context, implementation and mechanisms of impact.^50^ Semi-structured interviews will be undertaken with a subset of up to 20 participants (survivors and co-survivors), facilitators, and recruiters. Interviews will explore participant experiences after participating in the programme, and will consider perceived benefits or challenges, barriers to engagement and potential improvements. Facilitator interviews will focus on delivery, processes and experiences delivering the intervention. Recruiter interviews will focus on identification, screening and recruitment procedures, including challenges and alternative methods for participant identification or approach.

Interviews will be conducted remotely by telephone or video-call. Interview data will be digitally recorded, subject to the permission of each interview participant, pseudonymised, and transcribed verbatim. Data will be analysed using thematic analysis^51^ to identify patterns, and insights about intervention feasibility and acceptability. The analysis steps are described in Table 4. Data will be managed using NVivo software.

**Table 4:** Analysis steps for the process evaluation.^8^

**Step**	**Analysis activity**	**Action**
1	Familiarisation with the data	Immersion into the data through multiple read-throughs of the material to understand its content and context
2	Generating initial codes	Systematically identifying and labelling meaningful segments of data. These codes usually represent patterns, concepts, or themes that are present in the data
3	Searching for themes	Codes are grouped into broader themes which capture the essence of the data and reflect common patterns or ideas
4	Reviewing themes	Themes are critically examined to ensure they accurately represent the data and align with the research objective
5	Defining and naming themes	Themes are clearly defined, and a description is developed to capture their meaning
6	Producing a report	The synthesised findings are written which describes the themes, provides illustrative examples and discussing the implications of the findings

The findings of the qualitative work will supplement the descriptive statistics extracted as part of the study. They will contribute to the discussions at the end of the study, and form part of the synthesised results. The findings will help the team to explore and explain the overall experience of the CARESS intervention, and lessons learned from the feasibility study for a future randomised controlled trial. Where potential challenges, barriers or opportunity for improvement are identified, these findings will inform potential modifications to the programme content or study procedures to optimise intervention delivery, feasibility and acceptability for a future randomised controlled trial. These changes will be made upon conclusion of the feasibility study.

Group support sessions will be recorded, from which a purposively selected subset (10%) of recordings will be analysed using a checklist to assess fidelity. The qualitative approach described above will also help to understand what generated discussion within sessions, and what issues were discussed. Intervention fidelity will be assessed using the tenets highlighted by Mars et al.^38^

### Safety

An adverse event in this study is defined as any untoward medical occurrence in a participant receiving the complex intervention, which does not necessarily have a causal relationship with the intervention itself. Any adverse events related to CARESS will be recorded and disclosed to the study management team. Serious adverse events that have no relationship with the intervention (e.g., elective or pre-planned treatment for a pre-existing condition, or general care not associated with any deterioration in condition) will not be reported.

Causality and expectedness will be independently assessed by the facilitator, and separately by the CI with appropriate clinical input. All adverse events and serious adverse events will be documented within 24 h of the research team being made aware. Serious adverse events will be reported to the sponsor within 24 h, and any determined to be associated to participation in the intervention will be reported to the Research Ethics Committee panel within 15 days. Events will be followed up until the event has resolved or an outcome has been reached.

### Progression to a randomised controlled trial

Progression to a randomised controlled trial will be informed by evidence of the ability to recruit to our target sample size and successfully deliver the intervention. In previous research, minimum adherence has been defined as attendance to a single session.^52^ In CARESS, we define success as at least 75% of participants attending and completing the first group CARESS session, indicating minimum adherence. If engagement across the remaining sessions is below 75%, we will look closely at attendance patterns, and participant feedback to identify barriers. Intervention acceptability will be assessed by reviewing active participation with the intervention and by qualitative feedback on acceptability. Any modifications to our programme that will improve our processes, or delivery will be incorporated prior to progressing to a future randomised controlled trial.

### Patient and public involvement and engagement (PPIE)

The CARESS intervention was co-produced by an active PPIE group (six survivors, three co-survivors and an advocate). Two partners (PS, SM) are both co-applicants and are study management team members. PPIE partners co-led on a range of study initiatives including co-producing and facilitating workshops, creating intervention material and taking part in short videos to supplement group support sessions. They reviewed and influenced all patient facing material in the study and tested online data collection.

### Study oversight

A study management team chaired by the chief investigator has been established, and includes the study team, public partners, and co-investigators. The study management team meets monthly. A study steering committee includes five external experts and a survivor, providing light-touch oversight and guidance. This group meets at key study stages as defined by the steering committee. There is no independent data monitoring or formal oversight committee; these functions are fulfilled by the study management team and steering committee.

### Dissemination plans

Through active collaboration, we will disseminate study findings via multiple routes including academic publications, conference presentations, resuscitation councils (e.g., Resuscitation Council UK, European Resuscitation Council, and survivor organisations (e.g., SCA-UK).

### Data sharing

No data are available at this protocol stage. After study completion, datasets may be available upon reasonable request from Professor Kirstie Haywood (k.L.haywood@warwick.ac.uk) via a signed data-sharing agreement.

### Study status

At the time of submission, the CARESS intervention has been fully developed drawing together published evidence, systematic reviews and co-produced workshops involving survivors, co-survivors and health professionals. Intervention facilitators have been trained ready to deliver the intervention. Ethical approval was granted by North East – Newcastle & North Tyneside 2 Research Ethics Committee (24/NE/0135; September 27, 2024) and recruitment procedures have been agreed with participating sites. Participant-facing materials have been finalised following co-production with our PPIE partners. Recruitment launched June 20, 2025.

## Discussion

This innovative programme of research builds on the identified need to better support cardiac arrest survivors and co-survivors and sits within a steadily growing body of international research seeking to address this evidence gap.^22^, ^53^, ^54^, ^55^, ^56^ The research will impact healthcare through the collaborative development of an evidence-based and theory-informed intervention which has the potential to support long-term recovery, healing and survivorship following cardiac arrest for both survivors and co-survivors.

Impact will be realised through healthcare improvements for this growing and vulnerable population, achieved through the future implementation, in the first instance, into the NHS. However, enhancing the power of a digital platform suggests that wider dissemination across international boundaries is a real possibility.^57^ The study will contribute essential evidence towards the further enhancement of a minimum standard, post-hospital discharge care pathway that can be delivered to all cardiac arrest survivors and their families, thus maximising the chances of a good, long-term recovery.

However, cardiac arrest survivors are a heterogeneous group, ranging from those who experience minimal long-term health impairments, to those who suffer hypoxic brain injury and remain hospitalised.^2^, ^6^ In the UK, this approximates to 10% of survivors.^2^ The rehabilitation needs of cardiac arrest survivors who are unable to live independently are considerably different from those fit enough for discharge home.^2^, ^4^, ^58^ Those most severely injured will usually receive highly specialised care through rehabilitation consultants and neuro-rehabilitation physiotherapists, such that our planned intervention is unlikely to provide any additional benefit. Whilst this is an important area for future research,^4^, ^18^ the ability to engage with the intervention is an important inclusion criterion for the CARESS intervention.

In conclusion, this study will determine the feasibility and acceptability of delivering an online group psychosocial and exercise rehabilitation programme to both cardiac arrest survivors and co-survivors within an NHS setting. It will also provide invaluable evidence to confirm if the planned approach is suitable, generating data to facilitate the design of a randomised controlled UK multi-centre trial.

## Ethics declarations

Approval for the feasibility study was granted by North East – Newcastle & North Tyneside 2 (reference 24/NE/0135) Research Ethics Committee in September 2024. The study was prospectively registered (ISRCTN16382742).

## CRediT authorship contribution statement

**N.A. Pearson:** Writing – review & editing, Writing – original draft, Visualization, Resources, Project administration, Methodology, Conceptualization. **G. McGregor:** Writing – review & editing, Writing – original draft, Supervision, Resources, Methodology, Funding acquisition. **H. Sandhu:** Writing – review & editing, Writing – original draft, Methodology, Funding acquisition, Conceptualization. **K. Couper:** Writing – review & editing, Writing – original draft, Methodology, Funding acquisition, Conceptualization. **J. Bruce:** Writing – review & editing, Writing – original draft, Supervision, Methodology, Funding acquisition, Conceptualization. **P. Swindell:** Writing – review & editing, Writing – original draft, Funding acquisition, Conceptualization. **S. Menzies:** Writing – review & editing, Writing – original draft, Funding acquisition, Conceptualization. **D.R. Ellard:** Writing – review & editing, Writing – original draft, Methodology, Funding acquisition, Conceptualization. **R. Kandiyali:** Writing – review & editing, Writing – original draft, Supervision, Methodology, Conceptualization. **S. Ennis:** Writing – review & editing, Methodology, Conceptualization. **S. Patel:** Writing – review & editing, Methodology. **A. Hossain:** Writing – review & editing, Methodology, Funding acquisition. **K.L. Haywood:** Writing – review & editing, Writing – original draft, Supervision, Resources, Project administration, Methodology, Funding acquisition, Conceptualization. **Paul Swindell:** . **Stuart Menzies:** . **Jeanne Reilly:** . **Anne Brooks:** . **Dave Cleland:** . **Bernie Cleland:** . **Asad Kayani:** . **Rakhsana Yousaf:** . **John Long:** . **David Ellard:** .

## Funding

This research was funded by the National Institute for Health and Care Research (NIHR) Research for Patient Benefit Programme (NIHR204049). The views expressed are those of the author(s) and not necessarily those of the NIHR or the Department of Health and Social Care.

## Declaration of competing interest

KC is an associate editor of Resuscitation Plus and chair of the West Midlands region NIHR Research for Patient Benefit funding committee. The remaining authors report no conflicts of interest.
